# The Strabismus Opeation Made Easy

**Published:** 1865-06

**Authors:** 


The Strabismus Opeation Made Easy.—M. Emile Martin, of Marseilles, lately presented to the Academy of Medicine of Paris an instrument for quickly and securely dividing the tendon of the particular rectus muscle which is to be cut. The instrument is composed of a handle, to which is fixed a short stem ending in a blunt hook. In this hook lies a curved, very sharp blade, which is made to leave its recess in the hollow hook by pressure being made on a kind of spring fixed in the handle. The operation is thus considerably facilitated, and the section made completely as soon as the tendon has been hooked up.

